# Sex-specific expression of pheromones and other signals in gravid starfish

**DOI:** 10.1186/s12915-022-01491-0

**Published:** 2022-12-17

**Authors:** Mathias Jönsson, Marie Morin, Conan K. Wang, David J. Craik, Sandie M. Degnan, Bernard M. Degnan

**Affiliations:** 1 Centre for Marine Science, School of Biological Sciences, University of Queensland, Brisbane, QLD 4072 Australia; 2 Institute for Molecular Bioscience, ARC Centre of Excellence for Innovations in Peptide and Protein Science, The University of Queensland, Brisbane, QLD 4072 Australia

**Keywords:** *Acanthaster*, Aggregation, Broadcast spawning, Crown-of-thorns starfish, Echinodermata, Sea star, Spawning

## Abstract

**Background:**

Many echinoderms form seasonal aggregations prior to spawning. In some fecund species, a spawning event can lead to population outbreaks with detrimental ecosystem impacts. For instance, outbreaks of crown-of-thorns starfish (COTS), a corallivore, can destroy coral reefs. Here, we examine the gene expression in gravid male and female COTS prior to spawning in the wild, to identify genome-encoded factors that may regulate aggregation and spawning. This study is informed by a previously identified exoproteome that attracts conspecifics. To capture the natural gene expression profiles, we isolated RNAs from gravid female and male COTS immediately after they were removed from the Great Barrier Reef.

**Results:**

Sexually dimorphic gene expression is present in all seven somatic tissues and organs that we surveyed and in the gonads. Approximately 40% of the exoproteome transcripts are differentially expressed between sexes. Males uniquely upregulate an additional 68 secreted factors in their testes. A suite of neuropeptides in sensory organs, coelomocytes and gonads is differentially expressed between sexes, including the relaxin-like gonad-stimulating peptide and gonadotropin-releasing hormones. Female sensory tentacles—chemosensory organs at the distal tips of the starfish arms—uniquely upregulate diverse receptors and signalling molecules, including chemosensory G-protein-coupled receptors and several neuropeptides, including kisspeptin, SALMFamide and orexin.

**Conclusions:**

Analysis of 103 tissue/organ transcriptomes from 13 wild COTS has revealed genes that are consistently differentially expressed between gravid females and males and that all tissues surveyed are sexually dimorphic at the molecular level. This finding is consistent with female and male COTS using sex-specific pheromones to regulate reproductive aggregations and synchronised spawning events. These pheromones appear to be received primarily by the sensory tentacles, which express a range of receptors and signalling molecules in a sex-specific manner. Furthermore, coelomocytes and gonads differentially express signalling and regulatory factors that control gametogenesis and spawning in other echinoderms.

**Supplementary Information:**

The online version contains supplementary material available at 10.1186/s12915-022-01491-0.

## Background

Disparate marine invertebrates reproduce by broadcast spawning eggs and sperm into the water column, with fertilisation success often depending on the synchronised release of gametes [1–5]. Both gamete maturation and the timing of spawning within a population are often contingent upon the integration of exogenous physical and biological cues [6, 7]. This leads to a coordinated release of neuroendocrine factors, including hormones and neuropeptides internally and pheromones externally [8–11].

Many echinoderms—starfish, sea urchins, sea cucumbers and allies—are dioecious broadcast spawners that form seasonal aggregations before synchronously spawning [2, 3, 5, 12–19]. The high fecundity of some broadcast-spawning echinoderms, along with their resultant planktotrophic larvae, appears to contribute to their capacity to have localised outbreaks [17, 20–22], which in turn can negatively affect the ecology and resilience of marine ecosystems [23–25]. Understanding the internal and external signals that regulate the formation of aggregations and the timing of spawning, along with their receptors and signal transducers, may provide opportunities to control these events and prevent downstream population outbreaks [26–33].

The internal signalling systems regulating gametogenesis and spawning appear to be largely conserved in echinoderms, including in some species known for population outbreaks [34–43]. Seasonal changes in abiotic factors such as temperature, salinity, photoperiod and nutrient availability appear to influence the synchrony of gametogenesis and spawning through the activation of an internal signalling cascade that includes gonadotropin hormones [14, 44–56]. In asteroids (starfish), late-stage gametogenesis and gamete shedding appear to be regulated by the gonadotropin hormone relaxin-like gonad-stimulating peptide (RGP) [57–66]. RGP is produced primarily in the radial nerve and appears to interact with relaxin receptor-like G-protein-coupled receptors (GPCRs) expressed in ovarian follicle cells and testicular interstitial cells to induce spawning [66–71]. This induces follicle cells to secrete 1-methyladenine, which triggers the maturation of the oocytes and their dissociation from the follicle envelope [72–79].

In contrast to this understanding of echinoderm reproduction, the molecular mechanisms underlying the formation of aggregations and the timing of spawning events in the wild are understudied [80–87]. Male and female echinoderms can display different spawning behaviours, with males often initiating spawning events [80–87]. The crown-of-thorns starfish (COTS, *Acanthaster planci* species group) is one such species [88]. This corallivore is endemic to coral reefs throughout the Indo-Pacific and is a highly fecund, seasonal broadcast spawner that forms spawning aggregations during summer [89–92]. Abiotic cues, including photoperiod and water temperature, appear to regulate their aggregating in shallow waters [2, 90–93]. Males appear to secrete factors in the seminal fluid that can attract and induce spawning in other COTS [80, 86]. Their synchronised spawning can result in very high fertilisation success, leading to population outbreaks that, in turn, result in significant loss of live coral cover and ultimately ecosystem collapse [22, 85, 94–99]. Understanding the internal and external signals that regulate the formation of aggregations and the timing of spawning, along with their receptors and signal transducers, may provide opportunities to control these events and potentially downstream population outbreaks [26–28, 33].

Pacific COTS (*A. planci* cf. *A. solaris*) aggregating in captivity release a complex array of hundreds of proteins and other factors into the water column that attract other individuals, suggesting that pheromones contribute to the formation of spawning aggregations in nature [28]. The reception and processing of these water-borne signals are likely to occur via receptors on external-facing tissues and organs, including the sensory tentacles located at the distal end of each arm [100–102]. Here, we use a large-scale transcriptomics approach to identify factors contributing to reproductive aggregations and spawning. We have analysed multiple somatic tissues and organs, and gonads of gravid, wild COTS just prior to a mass spawning event. This approach is in contrast to most previous studies, which have focussed solely on gonads in animals translocated from their natural habitat [103–108]. We find that males and females differentially express putative attractants [28] in their gonads and chemosensory GPCRs and other receptors in their sensory tentacles. Females also upregulate a suite of neural signalling molecules in the sensory tentacles. Other neuroendocrine signalling factors involved in regulating reproduction and spawning in other echinoderms [34, 37–41, 46, 47, 49, 50, 103, 104, 108–113] are differentially expressed, including the unexpected upregulation of RGP in coelomocytes [57–66].

## Results

### Consistent and distinct gene expression profiles in tissues and organs from wild, gravid COTS

We generated 1.1 billion high-quality reads from 104 CEL-Seq2 transcriptomes from RNA isolated from eight tissues and organs removed from seven females and six males collected from the wild [114]. Tissues and organs were placed in RNALater within 2 h of the individual being dislodged from the reef, minimising transcriptional changes associated with translocation [115]. All COTS had large ripe gonads, with three individuals spawning upon collection (M. Jönsson and M. Morin, personal observation), suggesting that a natural spawning event was imminent [92]. One male radial nerve transcriptome was not further analysed because of a low mapping rate (< 0.5 million mapped reads). The remaining 103 transcriptomes had a 69.3% mapping rate to protein-coding sequences (CDS) in the GBR v1.1 genome (Additional file 1: Table S1.1) [28, 115]. A total of 18,032 of the 24,071 CDS were expressed in at least one tissue or organ, with each tissue/organ expressing on average 15,306 CDS (64% of the genome). The majority of all CDS (12,658 CDS; 71% of all expressed CDS) were detected in all tissues/organs.

**Fig. 1:**
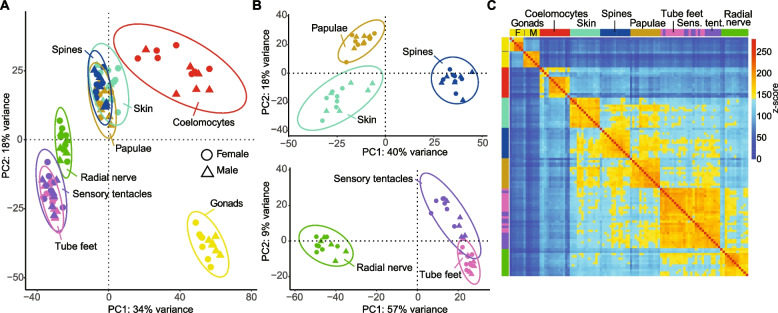
Differential expression of CDS in male and female COTS tissues/organs. **A** PCA of global gene expression profiles for the eight different COTS tissues/organs. **B** PCAs for only aboral (top) and oral (bottom) tissues/organs. **C** Pairwise correlation of eight tissue/organ transcriptomes; female and male gonads and coelomocyte transcriptomes are distinct from all other tissues. Pink, tube feet; purple, sensory tentacles; green, radial nerve; turquoise, skin; brown, papulae; blue, spines; red, coelomocytes; yellow, gonads; circles, females; triangles, males. Depicted values illustrate the scaled (*z*-score) expression levels based on collapsed VST-normalised read counts

Principal component analyses (PCAs) and hierarchical clustering of transcriptomes reveal strong clustering associated with tissue/organ type, regardless of sex, with the first principal component (PC1) accounting for 34% of the variation among samples (Fig. 1A). Gonad transcriptomes are distinct from all somatic tissues/organs, and the internally located coelomocytes are transcriptionally distinct from the two sets of external-facing tissues. The two clusters of external-facing tissues separate into oral tissues/organs, which face the substrate (radial nerve, sensory tentacles and tube feet) and aboral tissues/organs, which face the water column (papulae, skin and spines). Separate PCAs of oral and aboral transcriptomes distinguish each tissue/organ and indicate sensory tentacle, and tube feet transcriptomes are the most similar to each other (Fig. 1B). A global pairwise comparison of all transcriptomes is consistent with the PCAs and distinguishes male and female gonads from each other and the somatic tissues/organs (Fig. 1C).

The three oral tissues/organs uniquely upregulate 431 CDS (DESeq2 analyses; *p*-adj < 0.05) (Additional file 1: Table S1.2). These CDS are enriched in 83 GO terms consistent with the neural and chemosensory roles of these organs, such as ion channel and transmembrane signalling receptor activity (Additional file 1: Table S1.3). The three aboral tissues/organs have 382 significantly upregulated CDS (*p*-adj < 0.05) (Additional file 1: Table S1.4), which are enriched in eight GO terms consistent with these tissues/organs producing extracellular gene products (Additional file 1: Table S1.5). Coelomocytes have 1025 significantly upregulated CDS (Additional file 1: Table S1.6) enriched in 97 GO terms that reflect their diverse roles in metabolism, immunity and cell movement, including proteolysis and cytoskeletal organisation (Additional file 1: Table S1.7). The gonads have 2181 uniquely upregulated CDS (Additional file 1: Table S1.8) enriched in 423 GO terms, many of which are related to cellular metabolism and reproductive processes, including meiosis (Additional file 1: Table S1.9).

### COTS tissues and organs have sexually dimorphic gene expression

A comparison of gene expression in gravid male and female COTS using DESeq2 reveals 42% of the expressed CDS (7607) across all tissues/organs are significantly differentially expressed between males and females (Table 1; Additional file 1: Table S1.10). A majority of these (7448) are differentially expressed between the testes and the ovaries, with 4111 CDS upregulated in the testes. CDS upregulated in the testes and ovaries are significantly enriched in GO terms related to meiosis, and spermatogenesis and oogenesis, respectively. Doublesex and mab-3-related transcription factor 2 (DMRT2), kisspeptin and bindin are upregulated in the testes, while egg bindin receptors, egg coat matrix proteins and cyclin B are upregulated in the ovaries (Additional file 1: Table S1.10—S1.12).

**Table 1:** Number of significantly upregulated coding sequences

Tissue/organ	Total expressed CDS	Male	Female
Tube feet	15,611	0	3
Sensory tentacles	15,777	46	100
Radial nerve	14,712	11	7
Skin	15,282	0	7
Papulae	16,236	6	6
Spines	14,667	32	32
Coelomocytes	14,156	9	46
Gonads	16,004	4111	3337

All somatic tissues/organs surveyed in this study have sexually dimorphic gene expression profiles, with 283 CDS being significantly differentially expressed between sexes, including 14 transcription factors, 16 neuropeptides and five GPCRs (Tables 1 and 2; Additional file 1: Table S1.10). The sensory tentacles have the largest number of differentially expressed CDS (146), including the majority of the differentially expressed transcription factors, neuropeptides and GPCRs (Table 2).

**Table 2:** Sex-specific upregulated transcription factors, neuropeptides and GPCRs

CDS	Tissue/organ	Sex
**Transcription factors**		
Ladybird LBX1 (homeobox)	Sensory tentacles	Male
KLF5 (Kruppel C2H2 Zn-finger)	Sensory tentacles	Male
EGR1 (EGR C2H2 Zn-finger)	Sensory tentacles	Male
RORa (nuclear receptor)	Sensory tentacles	Male
Twist-related protein 2 (bHLH)	Sensory tentacles	Male
Scratch 1 (C2H2 Zn-finger)	Sensory tentacles	Male
ZNF423 (Kruppel C2H2 Zn-finger)	Sensory tentacles	Male
ZNF208 (Kruppel C2H2 Zn-finger)	Sensory tentacles	Male
SPDEF (ETS)	Spines	Female
CREB/ATF (bZIP)	Spines	Female
NR2E1 (nuclear receptor)	Spines	Male
NR2E3 (nuclear receptor)	Spines, radial nerve, papulae	Male
C/EBPa (bZIP)	Radial nerve	Male
c-Fos (bZIP)	Coelomocytes	Female
**Neuropeptides**		
AN peptide precursor protein	Sensory tentacles	Female
ApNP15a-like	Sensory tentacles	Female
ApNp22-like	Sensory tentacles	Female
ApNp23-like	Sensory tentacles	Female
ApNP26	Sensory tentacles	Female
ApNP27	Sensory tentacles	Female
ApNP29	Sensory tentacles	Female
ApNP31	Sensory tentacles	Female
Kisspeptin type precursor	Sensory tentacles	Female
Luqin-(LQ) type precursor	Sensory tentacles	Female
Orexin type precursor 2	Sensory tentacles	Female
Pedal peptide (PP) type precursor	Sensory tentacles	Female
Relaxin gonad-stimulating peptide	Sensory tentacles	Female
SALMFamide (L-type) precursor	Sensory tentacles	Female
Secretogranin 7B2-like protein	Sensory tentacles	Female
Starfish myorelaxant peptide	Sensory tentacles	Female
**GPCRs**		
Kisspeptin receptor 11.2	Sensory tentacles	Female
Somatostatin receptor type 2	Sensory tentacles	Female
Metabotropic glutamate receptor 8	Sensory tentacles	Male
Adhesion GPCR G2	Skin	Female
GPCR moody	Coelomocytes	Male

### Sex-biassed expression of putative attractants

**Fig. 2:**
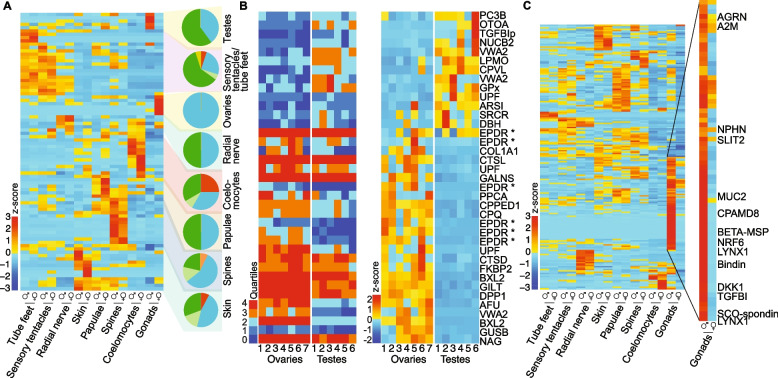
Expression of potential attractants and spawning factors in COTS tissues/organs. **A** Expression of 84 putative attractant CDS [28]. Male and female expression profiles are grouped by tissues/organs. Heatmap of expression levels in eight tissues/organs combine all analysed CEL-Seq2 data. Depicted values illustrate scaled (*z*-score) expression levels based on TPM normalised reads from seven females and six males (except radial nerve, which is from five males). Pie charts depict the proportion of highly expressed genes, per tissue, that fall into broad secreted protein classes (blue, hydrolytic enzymes; light green, other enzymes; orange, enzyme inhibitors; green, structural/signalling proteins; red, conserved uncharacterized proteins; yellow, uncharacterised proteins). **B** Individual COTS expression profiles of the 37 putative attractants that are significantly differentially expressed between the testes and the ovaries. CDS names are listed to the right. Left heatmap, quartile analysis of the mean transcript abundance; Q4, most highly expressed CDS; Q1, lowest expressed CDS; and 0, expression was not detected. Right heatmap, TPM normalised expression. Star (*) indicates the COTS-specific ependymin-related proteins. **C** Heatmap of 194 putative secreted proteins upregulated in male tissues/organs, 68 of which are highly expressed in the testes. Highlighted are conserved CDS of interest (Additional file 2: Table S2.3)

An aquarium-based study of Pacific COTS previously identified 94 proteins in an exoproteome that is secreted into seawater from non-gravid aggregating Pacific COTS containing conspecific chemoattractants [28]. Here, we find that 84 (89.4%) of the exoproteome CDS are expressed in gravid wild COTS (Fig. 2A), with 24 and 14 being significantly upregulated in females and males, respectively (Additional file 2: Table S2.1). All but one of these are differentially expressed between the ovaries and testes; beta-d-xylosidase 2 is upregulated in female coelomocytes (Fig. 2B). Among exoproteome CDS differentially expressed in the gonads, five of six differentially expressed ependymins are upregulated in the ovaries. Also, among the known exoproteins, the testes upregulate potential extracellular regulators, otoancorin and transforming growth factor-beta-induced protein ig-h3.

In addition to the exoproteome, we identified a further 194 genes encoding secreted factors that are significantly upregulated in male tissues/organs (Additional file 2: Table S2.2), of which 68 are most highly expressed in the testes (Fig. 2C; Additional file 2: Table S2.3). These include 33 conserved signalling and structural proteins, such as alpha-2-macroglobulin, bindin and beta-microseminoprotein, as well as 15 conserved uncharacterised proteins and two novel proteins.

### Female sensory tentacles upregulate a range of neural signalling molecules

**Fig. 3:**
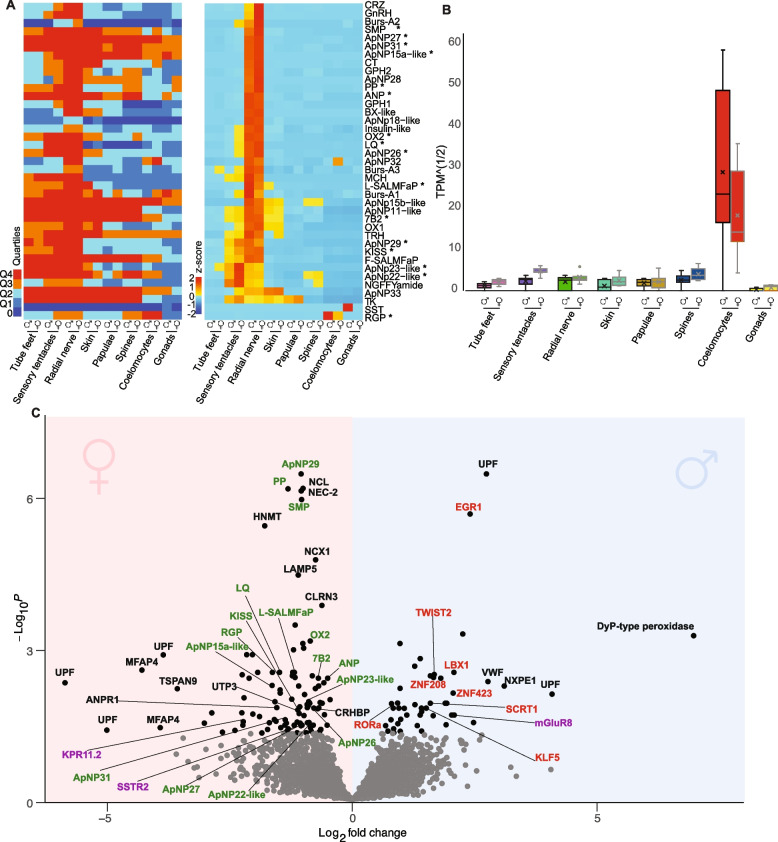
Neuropeptidome expression in the sensory tentacles and other tissues and organs. **A** Expression of 39 neuropeptides [122]. Male and female expression profiles are grouped by tissues/organs. Left heatmap, quartile analysis of the mean transcript abundance; Q4, most highly expressed CDS; Q1, lowest expressed CDS; and 0, no expression was detected. The right heatmap illustrates the scaled (*z*-score) expression levels based on TPM normalised read counts. Star (*) indicates the significant differential expression between male and female sensory tentacles. **B** Expression level of RGP across the different tissues/organs. Males and females are grouped by tissue/organ. Values depict square root-transformed TPM read counts of RGP. The expression of RGP is significantly higher in the coelomocytes of both sexes compared to all other tissues (*p*-adj < 0.001). **C** Volcanoplot of 15,777 expressed CDS in male and female sensory tentacles. Grey dots depict non-significantly differentially expressed CDS, and black dots depict significantly differentially expressed CDS (146) (*p*-adj < 0.05). Highlighted CDS names: green, neuropeptides; red, transcription factors; purple, GPCRs. CDS with negative and positive Log_2_fold change are upregulated in female and male sensory tentacles, respectively

Of the 39 neuropeptides expressed in wild gravid COTS, the vast majority are highly expressed in the oral sensory tissue/organs of both sexes, with the radial nerve expressing the most neuropeptides and at the highest levels (quartile 4, Fig. 3A; Additional file 3: Table S3.1). Notably, RGP is significantly upregulated in both male and female coelomocytes, compared to other tissues/organs (*p*-adj < 0.001) (Fig. 3B). A total of 23 neuropeptides are significantly differentially expressed between males and females, with 16 being upregulated in the female sensory tentacles (Fig. 3C; Additional file 3: Table S3.2), including neuropeptides with putative roles in reproduction such as kisspeptin, orexin and RGP, and several putative COTS-specific neuropeptides [57–63, 116–122].

An additional 130 CDS are significantly differentially expressed between male and female sensory tentacles, of which 100 are upregulated in females (Fig. 3C; Additional file 3: Table S3.3, Table S3.4). Among these are additional signalling molecules and receptors, including a cholecystokinin-type precursor and a corticotropin-releasing hormone-type precursor, a somatostatin receptor and an atrial natriuretic peptide receptor (Fig. 3C). Notably, no neuropeptides or hormones are significantly upregulated in the male sensory tentacles, despite upregulating a number of transcription factors (Table 2; Fig. 3C).

### Gravid COTS express a large suite of class A rhodopsin-like GPCRs

**Fig. 4:**
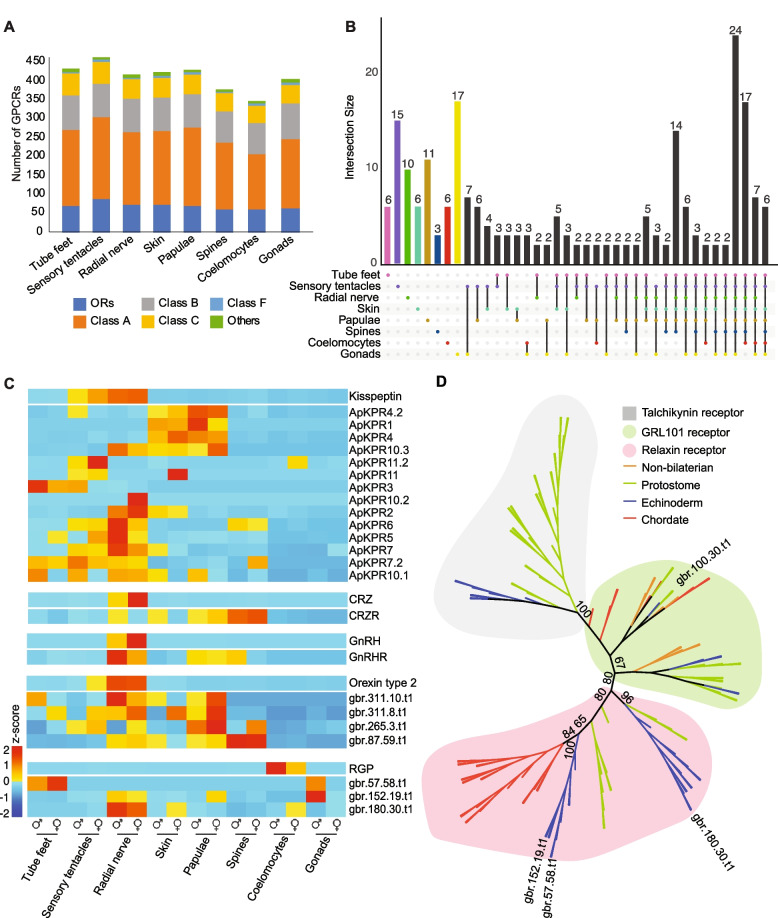
GPCR expression. **A** The number and subfamily type of GPCRs expressed in each tissue/organ (blue, olfactory receptors; orange, other class A rhodopsin-like; grey, class B secretin-like; yellow, class C glutamate-like; light blue, class F frizzled; green, others). **B** Upset plot of expressed GPCRs. The 276 GPCRs expressed commonly by all tissues are not shown. The interaction size is the number of GPCRs expressed uniquely or in tissue combinations shown at the bottom of the graph. **C** Expression heatmap of kisspeptin, CRZ, GnRH, orexin and RGP and their putative receptors. The expression of the neuropeptide is shown above their putative receptors. Male and female expression profiles are grouped by tissue/organ. Depicted values illustrate scaled (*z*-score) of TPM normalised read counts. **D** Maximum likelihood tree showing the phylogenetic relationship of four COTS relaxin receptor-likes to other metazoans. The outgroup is tachykinin-like receptors (grey). gbr.100.30.t1 is more closely related to GRL101 receptors (green), whereas gbr.180.30.t1, gbr.57.58.t1 and gbr.152.19.t1 cluster with other relaxin receptor-likes (red) and fall within echinoderm-specific clades. Model of substitution: LG + F + R8, with 1000 bootstrap replications visualised on the tree’s main branches (Fasta file and full tree with all bootstrap values are available in Additional files 9 and 10)

Wild gravid COTS express 559 GPCRs—59% of the total number of GPCRs in the genome (Additional file 4: Fig. S1; Additional file 5: Table S5.1)—of which 276 are expressed in all tissues. The sensory tentacles express the highest number of GPCRs (463), including the highest number of class A rhodopsin-like GPCRs (217), and putative olfactory receptors (87) (ORs) (Fig. 4A; Additional file 5: Table S5.2) [28]. The 15 GPCRs uniquely expressed in the sensory tentacles include eight ORs, alpha 1D adrenergic, dopamine and somatostatin receptor-likes (Fig. 4B; Additional file 5: Table S5.3).

Putative receptors of conserved neurohormones, including receptors for kisspeptin, gonadotropin-releasing hormone (GnRH), corazonin (CRZ), orexin and RGP are expressed in a wide range of tissues, including the coelomocytes (Fig. 4C). COTS express 15 kisspeptin receptors (ApKPRs), of which the majority are highly expressed in the radial nerve. Notably, female sensory tentacles are upregulating the kisspeptin precursor type and a putative cognate receptor (ApKPR11.2) compared to males (Fig. 4C; Additional file 6: Fig. S2). Putative receptors for CRZ and GnRH are lowly expressed, however appear to concentrate in the spines and radial nerve, respectively (Fig. 4C; Additional file 7: Fig. S3). Putative receptors for orexin are most highly expressed in the radial nerve, papulae and spines (Fig. 4C; Additional file 7: Fig. S3). Three putative RGP receptors are expressed in gravid COTS and appear to be echinoderm-specific (Fig. 4C, D). The putative receptors for RGP are lowly expressed in several tissues/organs; however, gbr.152.19.t1 is significantly upregulated in the testes compared to the ovaries (*p*-adj < 0.05) (Fig. 4C; Additional file 1: Table S1.10; Additional file 7: Fig. S3).

## Discussion

### Procuring wild transcriptomes

In this study, we analysed the transcriptional states of tissues and organs using replicated and defined biopsies from multiple gravid male and female COTS sampled directly on the Great Barrier Reef [114]. To our knowledge, this provides the first comprehensive sex-specific gene expression profiles in somatic tissues/organs and gonads of any echinoderm in the wild. The biologically replicated sampling enables the identification of genes that have consistent differences in the expression between tissues/organs and sexes. The high similarity of tissue transcriptomes between individuals that were collected over 7 days from two adjacent reefs suggests that the sampling regime has accurately captured native gene activity in wild COTS. Importantly, the sampling of COTS directly after their removal from the reef avoids transcriptional changes that may occur during translocation and being held in captivity. We have recently demonstrated that COTS translocated and kept in captivity exhibit large-scale and sustained transcriptional changes, with the expression of over 20% of the CDS in the genome changing significantly from the native expression profiles [115].

### Sexual dimorphism in crown-of-thorns starfish

Our analyses of the gene expression in both external and internal somatic tissues/organs, and in the testes and ovaries, provide new insights into the physiological and sensory states of this synchronous broadcast spawner [2]. For instance, we find that males predominantly upregulate secreted conspecific signalling factors in their testes, while females upregulate hormones and neuropeptides in their sensory tentacles. Most of the 94 exoproteins comprising a secretome previously shown to attract COTS [28] are upregulated in specific tissues, including the gonads, where 37 of these putative attractants are significantly differentially expressed between males and females. Moreover, RGP, a known gonadotropin and regulator of starfish reproduction [57–66], is significantly upregulated in the coelomocytes, suggesting a vital role for these cells in reproduction.

Although most echinoderm species are gonochoristic, there tend to be few visibly discernible external differences between sexes [123–126]. This is also the case for COTS [127, 128]. Nonetheless, males tend to spawn before females [80, 85, 86], indicating physiological and behavioural differences between the sexes. Our analysis of seven somatic tissues and organs, and gonads, uncovered sex-specific gene expression profiles in all tissues, with the gonads contributing 96.3% of the significant transcriptional differences between males and females. Within the gonads, 46.5% of all expressed CDS are significantly differentially expressed between sexes. The remaining 3.7% of sexually dimorphic gene expression is in somatic tissues and organs, consistent with gravid males and females having distinct physiologies that allow them to differentially respond to environmental and conspecific signals. In particular, the largest sex-specific differences in COTS appear in the most distal organs—sensory tentacles and spines—suggesting that males and females are most different in tissues/organs that are likely to first receive external molecular signals. There also appear to be sex-specific differences in internal physiology and signalling, indicated by sex-specific differences in coelomocyte gene expression. The differential spawning between sexes in other echinoderms [81, 83, 84, 87, 129] suggests that sexual dimorphisms, at least at the molecular level, are more widespread in this phylum than previously appreciated.

Differentially expressed CDS in COTS gonads include sex-specific proteins that are conserved in echinoderms. For instance, bindin—an acrosomal protein essential for sea urchin fertilisation—receptors for egg jelly, and guanylate cyclase are all upregulated in the testes [36–39, 109, 110, 113], and egg bindin receptor, egg coat matrix proteins and a putative maturation promoting factor are upregulated in the ovaries [34, 111, 112, 130–132]. In addition, DMRT-like transcription factor, kisspeptin and receptors for melatonin and thyrotropin-releasing hormone are upregulated in the testes. All of these appear to play a conserved role in sex determination and differentiation in deuterostomes and echinoderms [133–138].

### Sex-specific secretomes suggest a role in conspecific communication

The coordination of gamete maturation and spawning is essential for the high fertilisation success of most broadcast spawning marine invertebrates, including COTS [1–5]. Species with low-density populations, such as many echinoderms, often come together to form aggregations prior to spawning [19, 20, 139–141]. Both the aggregation and coordination of spawning appear to be mediated through the release of pheromones that are detected by conspecific chemosensory systems [80–87, 129]. Detection of released pheromones activates a cascade of internal signalling events that lead to physiological (e.g. gamete maturation) and behavioural (e.g. chemotaxis and synchronised spawning) changes [8, 10, 14, 51–79, 142, 143].

In aquaria, aggregations of COTS produce complex chemical plumes comprising hundreds of proteins that attract naive solitary adults [28]. Of the 94 characterised proteins in this plume, 84 are expressed in wild COTS, with 38 of these significantly differentially expressed between sexes. This suggests that the attractant proteins identified in the ex situ plumes may be playing a similar role in the wild. Among these are six ependymin-related proteins that are part of a COTS-specific expansion of this rapidly evolving gene family, which belongs to a larger metazoan ependymin family and is related to the developmental gene *LVN1.2* in the sea urchin *Lytechinus variegatus* [28, 144, 145]. These are strong candidates as conspecific signalling factors. Five ependymin-related proteins are upregulated in the ovaries and one in the testes. In addition to these characterised exoproteins, a large suite of secreted CDS is significantly upregulated in the testes, including a number of conserved factors such as alpha-2-macroglobulin and beta-microseminoprotein. Alpha-2-macroglobulin is a proteinase inhibitor that can bind many hormones [146–148], is present in seminal plasma [149] and appears to be involved in conspecific communication in the barnacle *Balanas amphitrite* [150]. Beta-microseminoprotein also appears to affect mating behaviours in the squid *Loligo pealeii* [151].

The presence of previously identified proteins in an exoproteome that attracts other COTS [28] and the identification of a raft of new secreted proteins, many of which are highly expressed in the testes, supports the premise that male and female COTS communicate their sexual identity and readiness to spawn through waterborne pheromones. Similar communication systems have been proposed for holothurians [87, 152, 153], ophiuroids [129], other asteroids [82] and other marine invertebrates [6, 151, 154, 155].

### Receptors and neuropeptides may regulate the response to external cues

Pheromones and other signals can affect conspecifics through the activation of receptors and downstream signalling pathways [156–159]. In wild COTS, we find that the sensory tentacles express the most GPCRs, including class A rhodopsin-like and putative olfactory receptors, consistent with their role as a chemosensory organ [28, 100–102]. The two class A rhodopsin-like GPCRs differentially expressed between male and female sensory tentacles are ApKPR11.2 and SSTR2. In addition, the sensory tentacles differentially express a large number of neuropeptides including orexin, which in mammals appears to regulate the sensitivity of expressed olfactory receptors to specific chemical signals [160–164]. Moreover, several of the highly expressed neuropeptides in COTS sensory tentacles and radial nerves have conserved roles in regulating reproduction in echinoderms and vertebrates. For instance, kisspeptin appears to regulate reproductive and metabolic pathways in holothurians [120] and the secretion of gonadotropin-releasing hormone (GnRH) in mammals [116, 117]. Mammalian kisspeptin appears to play a role in female-guided attraction towards males [118]. An expanded set of kisspeptin receptors (KPRs) were recently discovered in *Asterias rubens* and appear to interact with kisspeptin neuropeptides and SALMFamide peptides [165]. A functioning kisspeptin system has also been discovered in the sea cucumber *Apostichopus japonicus*, which appears to be seasonally expressed, indicating a potential role in reproduction [120]. There are at least 15 putative KPRs expressed in wild COTS. ApKPR1, 3, 8 and 9 are orthologues to KPRs in *A. rubens* that interact with kisspeptin. ApKPR6 and 7 are orthologues of *A. rubens* KPRs that interact with SALMFamide, which acts as a muscle relaxant in other starfish [166, 167]. The *A. rubens* orthologue of ApKPR11.2, which is upregulated in the COTS female sensory tentacles, has a low affinity for kisspeptin and SALMFamide [165]. The differential expression of KPRs, kisspeptin and SALMFamide precursors between male and female sensory tentacles suggests that these neuropeptide signalling systems are playing important roles in conspecific communication during reproduction.

COTS coelomocytes appear to be involved in transducing external signals to other tissues and organs, including the gonads. Specifically, we find that the coelomocytes upregulate RGP, which has a conserved role in regulating starfish reproduction [57–66]. To our knowledge, RGP has not been detected in other echinoderm coelomocytes. The coelomic fluid is a vital system for nutrient transport and immunity in echinoderms [168–170] and may also contain neuropeptides and other signals that regulate reproduction and other physiological states [171–174]. COTS coelomocytes express a large suite of class A rhodopsin-like GPCRs, including a putative corazonin (CRZ) receptor [175, 176], KPRs and thyrotropin-releasing hormone receptors. Thus, it appears coelomocytes are competent to receive internally secreted neuropeptides and hormones from the sensory tentacles and radial nerves and are playing a central role in regulating reproduction and spawning.

### Proposed regulation of COTS spawning based on gene expression in the wild

**Fig. 5:**
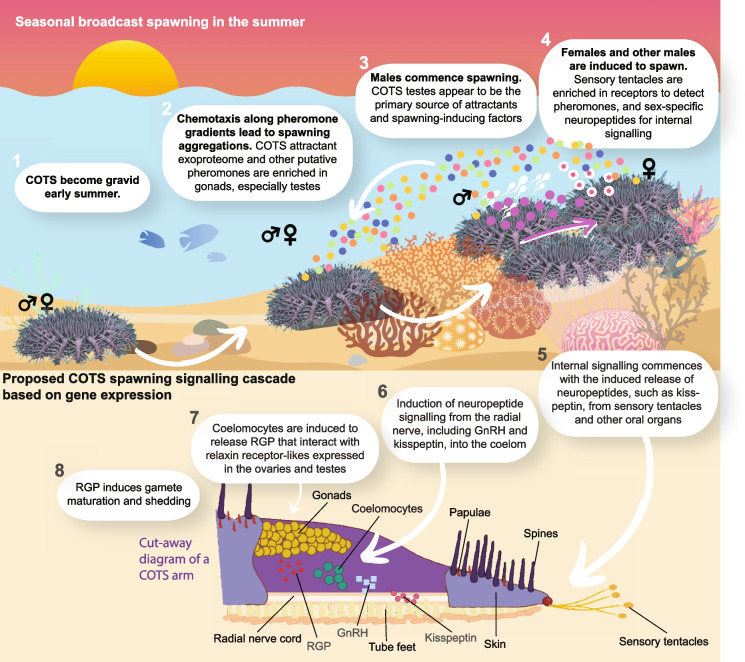
Proposed regulation of COTS spawning based on the gene expression profiles. (1) Physical seasonal environmental cues such as temperature and photoperiod trigger the commencement of gonad growth and gametogenesis and potentially sexually dimorphic gene expression. (2) Maturing individuals migrate towards shallow waters for spawning, with chemotaxis occurring along conspecific pheromone gradients. (3) Once aggregated, males initiate spawning based on abiotic and biotic (pheromones) cues. Spawning males release attractants and factors that trigger final oocyte maturation and spawning in females. (4) Females are competent to receive and transduce signals associated with the male spawn via receptors, hormones and neuropeptides in their sensory tentacles. (5) For both sexes, internal signalling cascades may include the secretion of neuropeptides, such as kisspeptin and SALMFamide, or a GnRH-like factor from sensory tentacles and radial nerves. (6) These may bind to cell-surface receptors on the coelomocytes, which may induce the secretion of RGP into the coelomic fluid. (7) RGP binds to cell-surface receptors on the gonads, (8) triggering the release of 1-methyladenine, which leads to oocyte maturation, the contraction of the gonad walls and the spawning of mature eggs

The tissue-, organ- and sex-specific gene expression profiles presented in this study were procured from wild, gravid COTS prior to a spawning event. A large number of putative attractant pheromones, spawning-inducing factors and neuropeptides and their cognate receptors are expressed in reproductive and sensory organs, suggesting their involvement in regulating COTS spawning. The testes appear to be the largest source of pheromones that could be released prior to, or at the time of, spawning (Fig. 5). Other putative pheromones appear to originate from the ovary and somatic tissues/organs of both sexes. The sensory tentacles appear to be the primary organ to detect exogenous signals, including pheromones, and to integrate these signals and promulgate information inwards. We posit that a combination of pheromones, receptors and internal signals, including conserved neurohormones and neuropeptides, allows for the detection of factors that guide the formation of aggregations and induce spawning (Fig. 5). Females activate a unique suite of receptors and signals consistent with each sex having a unique response to external cues.

We propose that exogenous signals received by the female sensory tentacles induce the release of kisspeptin, which is differentially upregulated in this organ. This may regulate the secretion of GnRH and/or CRZ from the radial nerve into the coelomic fluid, where they interact with receptors on the coelomocytes and, in turn, induce the release of RGP stores in these cells (Fig. 5). RGP may interact with RGP receptors in the gonads to regulate the last stages of gamete maturation and spawning, as in other asteroids [57–71]. To confirm this complete molecular cascade of events will require experimentation ex situ. Our transcriptome dataset on wild gravid COTS provides a framework to guide these future analyses.

## Conclusions

Analysis of tissue and organ transcriptomes that reflect the physiological state of gravid COTS around the time of spawning in the field suggests that there is extensive tissue-level sexual dimorphism beyond the gonads and that these differences play a role in COTS aggregation and spawning. Of particular note is the expression of an overabundance of putative pheromones in the testes, including many known to be secreted by aggregating COTS [28], and the expression of a large repertoire of receptors and signalling factors in the female sensory tentacles. These findings suggest mechanisms by which females could detect and respond to male spawning, through a signalling cascade that includes conserved reproductive neuropeptides and that ultimately induces oocyte maturation and female spawning.

## Methods

### Sampling of wild-caught COTS tissues

Seven gravid female and six gravid male COTS were collected from Davies and Lynch’s Reefs (18° 50′ S, 147° 39′ E, and 18° 76′ S, 147° 63′ E, respectively) on the Great Barrier Reef between 4 and 9 December 2019 and immediately transferred to aquaria containing ambient seawater onboard a vessel moored at the reef. All tissue and organ samples were dissected and placed in RNALater within 2 h of the COTS being removed from the reef. These were left in RNALater at 4 °C for 24 h and then stored at −20 °C.

Eight different tissues/organs—coelomocytes, gonads, sensory tentacles, tube feet, radial nerves, skin, papulae and spines—were sampled. Coelomocytes were separated from 1.5 ml of fluid extracted from the coelom using a syringe and 21-gauge needle by immediately centrifuging at 2000 × *g* at ambient temperature. The coelomic fluid was decanted, and coelomocytes were resuspended in RNALater [177]. The gonads were extracted via a small incision at the base of a random arm and gently removed via forceps. The sex of the animals was recorded, and the gonads were placed in RNALater. From each individual, (i) two sensory tentacles were dissected from the tip of a random arm, (ii) one tube foot was dissected from the middle of a random arm, (iii) a 30-mm length of the radial nerve cord was dissected from the middle of a random arm, (iv) the skin was dissected from the proximal base of a random arm, (v) approximately 50 papulae were dissected from the dissected skin samples while in RNALater (the papulae-less skin was used to make the skin libraries) and (vi) the spine samples consisted of the epithelium layer scraped off of one spine, collected from the base of a random arm.

### CEL-Seq2 library construction and sequencing

RNA from each individual tissue/organ was extracted using TRI Reagent (Sigma) following the manufacturer’s protocol. The quantity and quality of the RNA were assessed using a Qubit® fluorometer (Invitrogen-Life Technologies) and the Agilent 2100 Bioanalyzer (Agilent Technologies). CEL-Seq2 library construction and sequencing were performed as described in Hashimshony et al. [178] with each individual sample being barcoded. The eight pooled tissue/organ libraries were sequenced on the Illumina HiSeq X ten platform.

Raw sequencing reads were assessed for quality and adaptor contents using FastQC [179]. Reads were then analysed using a CEL-Seq2 pipeline publicly available on GitHub (https://github.com/yanailab/CEL-Seq-pipeline; version 1.0) [178, 180]. Reads were trimmed to 35 bp and demultiplexed, then mapped to the Great Barrier Reef *Acanthaster planci* genome [28] using Bowtie2. Transcript counts of each GBR v1.1 CDS [115] were generated using HTSeq [181]. Samples with mapped transcripts < 0.5 million were discarded as low-quality [182].

### Differential gene expression analyses

To remove potential technical read errors often associated with lowly expressed genes [183], we tested different expression threshold levels of an average of ≥ 0.25 and ≥ 1 read per gene per library for all replicates of a given tissue and opted for the expression threshold of ≥ 0.25 mean reads per tissue/organ (Additional file 8: Fig. S4). DESeq2 [184] was performed to identify differentially expressed CDS with a *p*-adjusted (*p*-adj) value of < 0.05. Gene Ontology (GO) enrichment analyses of differentially expressed CDS were performed using Fisher’s exact test function available on Omicsbox (v1.4.11), using the GO annotation provided by Blast2GO. GO terms were considered enriched with a false discovery rate (FDR) value of < 0.05. Principal component analyses (PCAs) were used to visualise the differences in gene expression between tissues/organs and sexes. PCAs were performed in RStudio on variance-stabilise transformed (VST) counts obtained with DESeq2. Heatmaps were created to visualise the expression patterns of genes of interest, using transcripts per million (TPM) normalised raw reads, in the R package “pheatmap” [185, 186]. TPM reads were also used to calculate the quartile values for each individual. Expressed genes were classified into four quartiles ranging from lowest (Q1; bottom 25%) to highest (Q4; top 25%) level of transcript abundance. Upset plots were used as an alternative to Venn diagrams, to illustrate the eight-way expression of GPCRs between the eight different tissues. These upset plots were constructed using the R package UpsetR [187]. All analyses and visualisations were performed in RStudio version 4.0.2 [188].

### Identification of secreted factors

The COTS v1.1 gene models (GBR v1.1) [115] were screened for genes encoding signal peptides using SignalP 5.0 [189] and then for transmembrane domains using TMHMM Server v. 2.0 [190, 191]. Proteins were considered secreted if they possessed a predicted signal peptide but no transmembrane domain. CDS were blasted on NCBI using blastp, and sequences with no significant matches (*E*-value < 0.05) were considered potentially novel species-specific proteins.

### Identifying neuropeptides and putative cognate receptors in the COTS genome

The neuropeptidome of COTS has been characterised [122]. Gonadotropin-releasing hormone (GnRH) and corazonin (CRZ) gene models were annotated by blasting previously identified GnRH and CRZ sequences [122] to the COTS genome [28, 115]. We visualised and predicted the location of the GnRH and CRZ by identifying mapped reads using the IGV genome browser (v.2.8.6) [192]. The positions were as follows: GnRH (gbr.55.97_1.t1; scaffold55, position 1,659,123–1,676,800) and CRZ (gbr.55.96_1.t1; scaffold55, position 1,578,255–1,603,383). Putative peptide receptors were identified via Blast2GO annotation and blasting previously characterised GnRH-type and CRZ-type receptors [175, 176] to the COTS genome. Putative kisspeptin receptors (KPRs) were previously identified and deorphanised in *Asterias rubens* [165], and 15 orthologous genes were identified in COTS (ApKPR1-11). Additional KPR7-like (ApKPR7.2) and KPR11-like (ApKPR11.2) were identified by phylogenetic analysis using modified methods from Escudero Castelán et al. [165] and FastTree2 [193] (Additional file 6: Fig. S2). Putative receptors of RGP have previously been described in COTS [70]. To further assess the sequence similarities of these relaxin receptor-like sequences, we blasted the NCBI protein database (blastp) [194] and chose sequence hits from a diversity of metazoans to construct multiple sequence alignments using MAFFT v.7.455 [195] with manual editing in AliView v1.26 [196]. The phylogenetic relationships of the COTS relaxin receptor-like GPCRs were identified in an unrooted maximum likelihood tree constructed in IQ-TREE v.1.6.12 [197]. The best-fit model of substitution was identified by IQ-TREE as LG + F + R8. We used 1000 bootstrap replications. A Fasta file containing all sequences used for tree inference can be found in Additional file 9.

## Supplementary Information

**Additional file 1:** **Table S1.1.** Transcriptome statistics (read counts and mapping). **Table S1.2.** Significantly upregulated CDS in oral tissues/organs. **Table S1.3.** GO terms significantly enriched in oral tissues/organs. **Table S1.4.** Significantly upregulated CDS in aboral tissues/organs. **Table S1.5:** GO terms significantly enriched in aboral tissues/organs. **Table S1.6.** Significantly upregulated CDS in the coelomocytes. **Table S1.7.** GO terms significantly enriched in the coelomocytes. **Table S1.8.** Significantly upregulated CDS in the gonads. **Table S1.9.** GO terms significantly enriched in the gonads. **Table S1.10.** Significantly differentially expressed genes between males and females. **Table S1.11.** GO terms significantly enriched in the testes. **Table S1.12.** GO terms significantly enriched in the ovaries.

**Additional file 2:** **Table S2.1.** Aggregation-inducing exoproteome CDS differentially expressed between males and females. **Table S2.2.** Significantly upregulated secreted CDS in male tissues/organs. **Table S2.3.** 68 secreted CDS that are upregulated in males and highly expressed in the testes.

**Additional file 3:** **Table S3.1.** Expressed neuropeptides. **Table S3.2.** Significantly differentially expressed neuropeptides between males and females. **Table S3.3.** Significantly differentially expressed CDS between male and female sensory tentacles. **Table S3.4. **DeSeq2 results of 15,777 expressed CDS in male and female sensory tentacles.

**Additional file 4:** **Fig. S1.** GPCR expression. Expression heatmap of 559 expressed GPCRs. Male and female expression profiles are grouped by tissues/organs. Pie charts depict the proportion of highly expressed GPCRs per tissue that fall into specific subclasses (Fig. 4A).

**Additional file 5:** **Table S5.1. **Expressed GPCRs. **Table S5.2. **Expressed GPCRs in the sensory tentacles. **Table S5.3. **Uniquely expressed GPCRs in the sensory tentacles.

**Additional file 6:** **Fig. S2.** Phylogenetic analysis of bilaterian kisspeptin receptor-likes. FASTA sequences were used from Escudero Castelán et al. [165], with the addition of GPCR-54-likes from the GBR v1.1 COTS genome. The tree was rooted with galanin/allostatin A-type receptors as an outgroup as per Escudero Castelán et al. [165]. Clade 1 includes ApKPR2-4 and chordate kisspeptin receptor-likes. Branch support values are depicted at each node (Shimodaira-Hasegawa test). Arrowheads depict 14 COTS kisspeptin-type receptors (ApKPR1-11) (ApKP 8 & 9 were not included in the analysis due to missing transmembrane regions). The scale bar represents the estimated proportional sequence divergence. Receptors in red text indicate ligands that have been identified experimentally as per Escudero Castelán et al. [165].

**Additional file 7:** **Fig. S3. **Expression of neuropeptides and putative cognate receptors. Quartile analysis (left); red, top 25% highest expressed CDS (Q4); and dark blue, gene is not expressed (0); and TPM normalised gene expression heatmap (right) of kisspeptin, CRZ, GnRH, orexin, RGP and putative cognate receptors.

**Additional file 8:** **Fig. S4.** Comparison of different expression threshold values. **A** PCA of all individual transcriptomes separated into respective tissue cluster (colour) and sex (shape). The threshold value of expression was set to a mean number of ≥ 0.25 reads per tissue. **B** Same PCA as (**A**) but here the threshold value was set to a mean number of ≥ 1 reads per tissue. This comparison reveals a small difference between the two threshold values. (**A**) resulted in 18,032 CDS genes counted as expressed and (**B**) resulted in 16,127 CDS genes counted as expressed. Due to the replicated sampling, we opted to include as many CDS as possible in the differential expression analysis.

**Additional file 9:** Fasta file of sequences used to infer RGP receptor phylogeny.

**Additional file 10:** Phylip tree file of Fig. 4D.

## Data Availability

The raw RNA sequences generated in this study are publicly available in the NCBI Sequence Read Archive (SRA) [198], under BioProject PRJNA821257 [114]. GBR v1.1 gene models and transcriptomes can be visualised at https://apollo-portal.genome.edu.au/degnan/cots.

## Abbreviations

COTS: Crown-of-thorns starfish
RGP: Relaxin-like gonad-stimulating peptide
GPCRs: G-protein-coupled receptors
CDS: Coding sequences
PCA: Principal component analysis
PC1: First principal component
DMRT2: Doublesex and mab-3 related transcription factor 2
ORs: Olfactory receptor-likes
ApKPR: *Acanthaster planci* Kisspeptin receptor
KPR: Kisspeptin receptor
GnRH: Gonadotropin-releasing hormone
CRZ: Corazonin
GO: Gene Ontology
FDR: False discovery rate
VST: Variance-stabilise transformed
TPM: Transcripts per million
