# Supplementary material for: Sex-specific expression of pheromones and other signals in gravid starfish
Source: BMC Biol. 2022 Dec 17;20:288. doi: 10.1186/s12915-022-01491-0 (PMC9759900; doi:10.1186/s12915-022-01491-0)
Supplement: Supplementary file 4 — Additional file 4: Fig. S1. GPCR expression. Expression heatmap of 559 expressed GPCRs. Male and female expression profiles are grouped by tissues/organs. Pie charts depict the proportion of highly expressed GPCRs per tissue that fall into specific subclasses (Fig. 4A). [file 12915_2022_1491_MOESM4_ESM.pdf]

Fig. S1

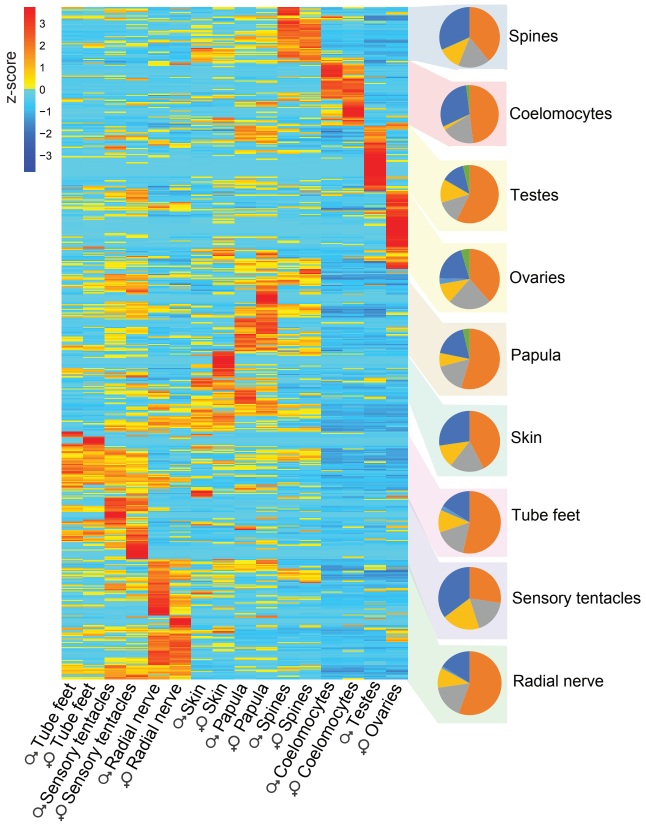

Fig. S1: GPCR expression. Expression heatmap of 559 expressed GPCRs. Male and female expression profiles are grouped by tissues/organs. Pie charts depict the proportion of highly expressed GPCRs per tissue that fall into specific subclasses (Fig. 4A).
