# Supplementary figures and images for: Sex-specific expression of pheromones and other signals in gravid starfish
Source: BMC Biol. 2022 Dec 17;20:288. doi: 10.1186/s12915-022-01491-0 (PMC9759900; doi:10.1186/s12915-022-01491-0)

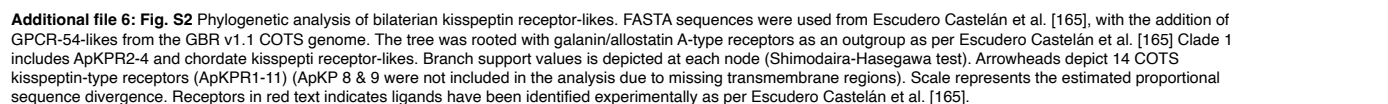

Supplement: Supplementary file 6 — Additional file 6: Fig. S2. Phylogenetic analysis of bilaterian kisspeptin receptor-likes. FASTA sequences were used from Escudero Castelán et al. [165], with the addition of GPCR-54-likes from the GBR v1.1 COTS genome. The tree was rooted with galanin/allostatin A-type receptors as an outgroup as per Escudero Castelán et al. [165]. Clade 1 includes ApKPR2-4 and chordate kisspeptin receptor-likes. Branch support values are depicted at each node (Shimodaira-Hasegawa test). Arrowheads depict 14 COTS kisspeptin-type receptors (ApKPR1-11) (ApKP 8 & 9 were not included in the analysis due to missing transmembrane regions). The scale bar represents the estimated proportional sequence divergence. Receptors in red text indicate ligands that have been identified experimentally as per Escudero Castelán et al. [165]. [file 12915_2022_1491_MOESM6_ESM.pdf]
