# Supplementary material for: Sex-specific expression of pheromones and other signals in gravid starfish
Source: BMC Biol. 2022 Dec 17;20:288. doi: 10.1186/s12915-022-01491-0 (PMC9759900; doi:10.1186/s12915-022-01491-0)
Supplement: Supplementary file 7 — Additional file 7: Fig. S3. Expression of neuropeptides and putative cognate receptors. Quartile analysis (left); red, top 25% highest expressed CDS (Q4); and dark blue, gene is not expressed (0); and TPM normalised gene expression heatmap (right) of kisspeptin, CRZ, GnRH, orexin, RGP and putative cognate receptors. [file 12915_2022_1491_MOESM7_ESM.pdf]

Fig. S3

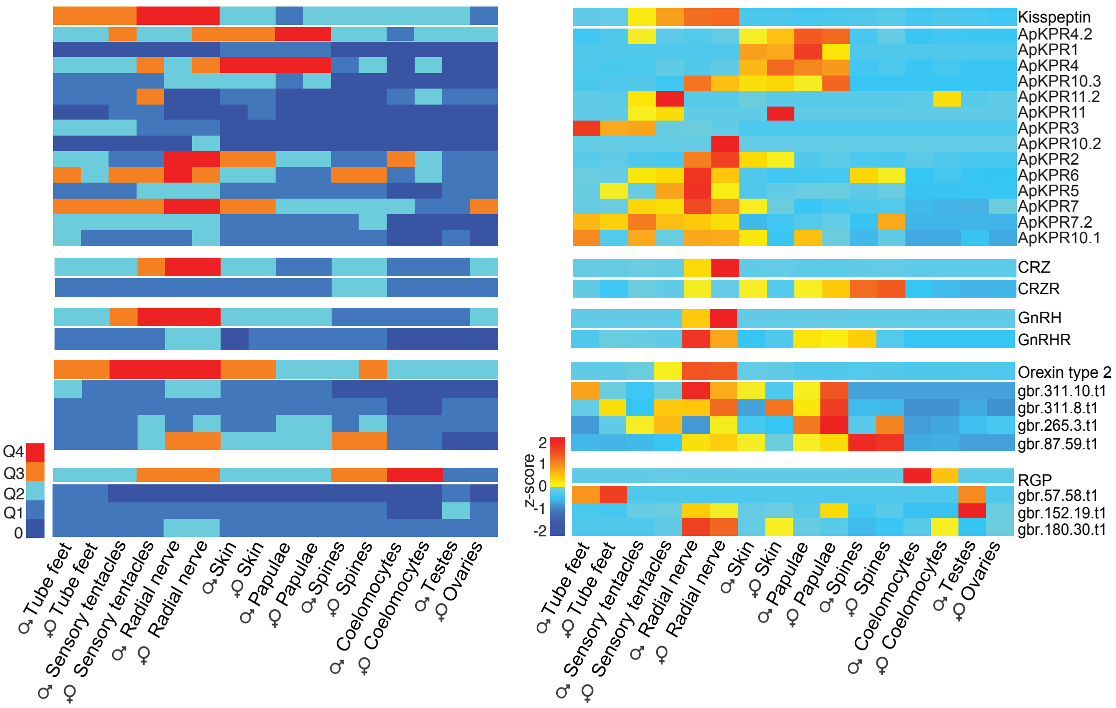

Fig. S3: Quartile analysis (left); red, top 25% highest expressed CDS (Q4); and dark blue, gene is not expressed (0); and TPM normalised gene expression heatmap (right) of kisspeptin, CRZ, GnRH, orexin, RGP and putative cognate receptors.
