# Supplementary material for: Sex-specific expression of pheromones and other signals in gravid starfish
Source: BMC Biol. 2022 Dec 17;20:288. doi: 10.1186/s12915-022-01491-0 (PMC9759900; doi:10.1186/s12915-022-01491-0)
Supplement: Supplementary file 8 — Additional file 8: Fig. S4. Comparison of different expression threshold values. A PCA of all individual transcriptomes separated into respective tissue cluster (colour) and sex (shape). The threshold value of expression was set to a mean number of ≥ 0.25 reads per tissue. B Same PCA as (A) but here the threshold value was set to a mean number of ≥ 1 reads per tissue. This comparison reveals a small difference between the two threshold values. (A) resulted in 18,032 CDS genes counted as expressed and (B) resulted in 16,127 CDS genes counted as expressed. Due to the replicated sampling, we opted to include as many CDS as possible in the differential expression analysis. [file 12915_2022_1491_MOESM8_ESM.pdf]

Fig. S4

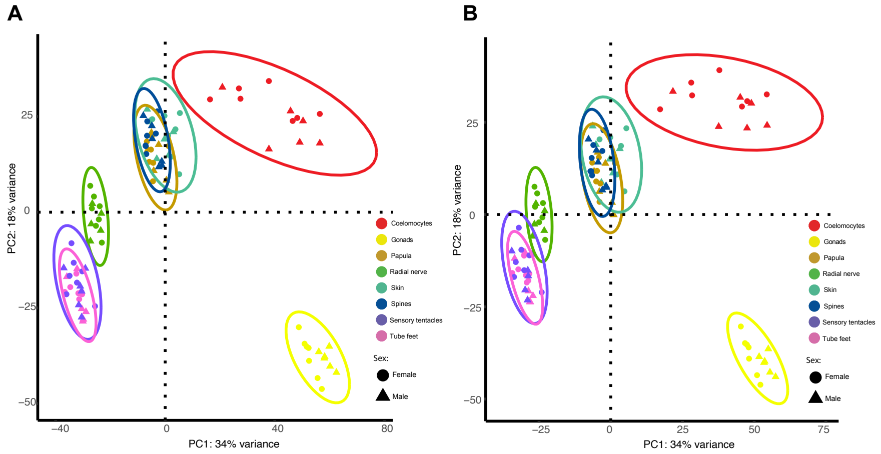

Fig. S4: Comparison of different expression threshold values. A PCA of all individual transcriptomes separated into respective tissue cluster (colour) and sex (shape). The threshold value of expression was set to a mean number of ≥ 0.25 reads per tissue. B Same PCA as (A) but here the threshold value was set to a mean number of ≥ 1 reads per tissue. This comparison reveals no discernible difference between the two threshold values. However (A) resulted in 18,032 CDS genes counted as expressed whereas (B) resulted in 16,127 CDS genes counted as expressed. Due to the replicated sampling, we opted to include as many CDS genes as possible in the differential expression analysis.
